# Gluten quantification in gluten-free food for celiac people in Lombardy and Emilia Romagna - Italy

**DOI:** 10.1093/eurpub/ckac131.237

**Published:** 2022-10-25

**Authors:** E Pavoni, B Bertasi, G Finazzi, V Filipello, M-N Losio

**Affiliations:** 1 Food Safety Department, IZSLER, Brescia, Italy; 2 NRC for Emerging Risks in Food Safety, IZSLER, Brescia, Italy

## Abstract

**Background:**

In recent decades, the celiac disease showed a gradual increase in prevalence. Therefore, there was a raised demand of gluten-free products. EU Reg. 1169/2011 states that 20 mg.kg-1 (ppm) is the maximum gluten content in food for celiac people, and that “gluten-free” labelling must be put on compliant food packages. This work is a study on different food categories, aiming at verifying the safety of analyzed samples.

**Methods:**

Totally, 4615 gluten-free-labelled specimens were collected from January 2019 to April 2022 (pasta, 2944; cured meat, 566; flours/bakery, 489; sweets, 125 and other matrices, 491). A commercially available E.L.I.S.A. kit, according to the AOAC 2012.01-2016 method, was used to quantify gluten.

**Results:**

In 97% of samples (4475) the gluten content was <5 ppm (lower LOD), and in 2.4% (112) it was between 5 ppm and <20 ppm. In the remaining 0.6% (28), the gluten concentration was ≥20 ppm. Of these, 0.32% (15) were between 20 and <80 ppm (upper LOD), and 0.28% (13) ≥80 ppm.

**Conclusions:**

The increased prevalence of celiac disease and the consumers’ perception that a gluten-free diet gives benefits, lead to a greater demand of gluten-free products. In this study, 99.4% of samples were compliant with the gluten-free labelling and safe for celiac consumers. The 0.32% had a gluten content between 20 and 80 ppm, still considered “compliant”, according to the EU Reg. 828/2014 that defines as very low gluten containing (thus edible for some celiac groups), those products with a gluten content <100 ppm. Only 0.28% of samples was non-compliant (≥80 ppm). However, the authors accounted irregular those foods with ≥20 ppm. Considering the importance of these products in the daily diet, and the increasing probability to get sick by individuals, the study of their compliance to the law limits results to be important.

**Key messages:**

• A continuous surveillance of gluten-free-labelled food products is very important to prevent risks for celiac consumers.

• The foodstuffs distributed in the two considered regions are mainly safe for celiac patients.

